# Development of Piperine-Loaded Solid Self-Nanoemulsifying Drug Delivery System: Optimization, In-Vitro, Ex-Vivo, and In-Vivo Evaluation

**DOI:** 10.3390/nano11112920

**Published:** 2021-10-31

**Authors:** Ameeduzzafar Zafar, Syed Sarim Imam, Nabil K. Alruwaili, Omar Awad Alsaidan, Mohammed H. Elkomy, Mohammed M. Ghoneim, Sultan Alshehri, Ahmed Mahmoud Abdelhaleem Ali, Khalid Saad Alharbi, Mohd Yasir, Kaveripakkam M. Noorulla, Sami I. Alzarea, Abdullah S. Alanazi

**Affiliations:** 1 Department of Pharmaceutics, College of Pharmacy, Jouf University, Sakaka 72341, Al-Jouf, Saudi Arabia; Nkalruwaili@ju.edu.sa (N.K.A.); osaidan@ju.edu.sa (O.A.A.); Mhalkomy@ju.edu.sa (M.H.E.); 2 Department of Pharmaceutics, College of Pharmacy, King Saud University, Riyadh 11451, Saudi Arabia; salshehri1@ksu.edu.sa; 3 Department of Pharmacy Practice, College of Pharmacy, AlMaarefa University, Ad Diriyah 13713, Saudi Arabia; mghoneim@mcst.edu.sa; 4 Department of Pharmaceutics and Industrial Pharmacy, College of Pharmacy, Taif University, Taif 21944, Saudi Arabia; a.mali@tu.edu.sa; 5 Department of Pharmacology, College of Pharmacy, Jouf University, Sakaka 72341, Al-Jouf, Saudi Arabia; kssalharbi@ju.edu.sa (K.S.A.); samisz@ju.edu.sa (S.I.A.); 6 Department of Pharmacy, College of Health Sciences, Arsi University, Asella P.O. Box 396, Ethiopia; mohdyasir31@gmail.com (M.Y.); my_pharma31@yahoo.com (K.M.N.); 7 Department of Clinical Pharmacy, College of Pharmacy, Jouf University, Sakaka 72341, Al-Jouf, Saudi Arabia; Asdalananzi@ju.edu.sa; 8 Health Sciences Research Unit, Jouf University, Sakaka 72341, Al-Jouf, Saudi Arabia

**Keywords:** oral delivery, piperine, solid self nanoemusifying, antimicrobial activity, antihypertensive activity

## Abstract

Hypertension is a cardiovascular disease that needs long-term medication. Oral delivery is the most common route for the administration of drugs. The present research is to develop piperine self-nanoemulsifying drug delivery system (PE-SNEDDS) using glyceryl monolinoleate (GML), poloxamer 188, and transcutol HP as oil, surfactant, and co-surfactant, respectively. The formulation was optimized by three-factor, three-level Box-Behnken design. PE-SNEDDs were characterized for globule size, emulsification time, stability, in-vitro release, and ex-vivo intestinal permeation study. The optimized PE-SNEDDS (OF3) showed the globule size of 70.34 ± 3.27 nm, percentage transmittance of 99.02 ± 2.02%, and emulsification time of 53 ± 2 s Finally, the formulation OF3 was transformed into solid PE-SNEDDS (S-PE-SNEDDS) using avicel PH-101 as adsorbent. The reconstituted SOF3 showed a globule size of 73.56 ± 3.54 nm, PDI of 0.35 ± 0.03, and zeta potential of −28.12 ± 2.54 mV. SEM image exhibited the PE-SNEDDS completely adsorbed on avicel. Thermal analysis showed the drug was solubilized in oil, surfactant, and co-surfactant. S-PE-SNEDDS formulation showed a more significant (*p* < 0.05) release (97.87 ± 4.89% in 1 h) than pure PE (27.87 ± 2.65% in 1 h). It also exhibited better antimicrobial activity against *S. aureus* and *P. aeruginosa* and antioxidant activity as compared to PE dispersion. The in vivo activity in rats exhibited better (*p* < 0.05) antihypertensive activity as well as 4.92-fold higher relative bioavailability than pure PE dispersion. Finally, from the results it can be concluded that S-PE-SNEDDS might be a better approach for the oral delivery to improve the absorption and therapeutic activity.

## 1. Introduction

Hypertension is a disease associated with high blood pressure that leads to serious impediments like a high risk of heart disease, stroke, as well as maybe death [1]. Many synthetic therapeutic agents are available in the market for the treatment of hypertension but have side effects that may be fatal for other organs. Nowadays, natural bioactive therapeutic agents are in demand to cure different diseases (diabetes, hypertension, arthritis, and cancer) due to lesser side effects than synthetic molecules [2]. The oral route is the most prominent route for the administration of drugs. However, the problems like major inter-subject variability, dose fluctuation, and low bioavailability are associated with poorly/sparingly soluble therapeutics. Approximately 40% of therapeutic agents have reported low aqueous solubility, which leads to low bioavailability [3,4].

Piperine (PE) is a naturally occurring bioactive molecule of piper nigrum. Chemically, it belongs to alkaloids (a weak base) and is reported for different pharmacological activities like anti-hypertensive, antidiabetic, anticancer, antimicrobial, anti-inflammatory, and antioxidant activity [5,6,7,8]. It is a poorly soluble drug (40 µg/mL, log *p* = 2.25) and offers low bioavailability due to poor dissolution, which is a rate-limiting step for absorption [9]. Various researches have reported the PE formulation for the enhancement of solubility, bioavailability, and therapeutic efficacy. Zafar et al., formulated PE nanosuspension using HPMC as a polymer by the nanoprecipitation method. The prepared nanosuspension showed better dissolution and 3.65-fold higher bioavailability than pure PE dispersion [10]. In another research, Ray et al. formulated amphotericin B and piperine-loaded eudragit-guar gum nanoparticles for antileishmanial activity. The prepared nanoparticles depicted controlled drug release with improved oral bioavailability as well as about 96% inhibition of Leishmania parasites [11].

To overcome these limitations, various lipid-based nano-formulations were previously reported for the enhancement of solubility, bioavailability, and oral efficacy. The delivery systems like solid lipid nanoparticles [12], nanostructure lipid carriers [13], liposomes [14], niosomes [15], and self-nanoemulsifying drug delivery systems [16,17] have shown the potential for the enhancement of solubility and bioavailability. Among these, SNEDDS is the most prominent and novel formulation approach for the oral delivery of poorly soluble therapeutics. It is considered as the isotropic mixture of drug, liquid lipid, surfactant, and cosurfactant. With oral administration, SNEDDS comes in contact with stomach fluid and is automatically self-emulsified with globule size <100 nm in peristalsis movement, which leads to solubilization of the drug, increases the gastric stability, and absorbs directly through the lymphatic way, reducing the first-pass effect, and hence increasing the bioavailability. However, there is a compatibility issue with hard/soft gelatin capsule; SNEDDS transformed into solid SNEDDS (S-SNEDDS) to improve the patient compliance, ease of production, scale-up, and transportation [18].

Therefore, the objective of this research was to develop and optimize PE-SNEDDS by Box-Behnken design (BBD) and evaluate for globule size, emulsification time, and percentage transmittance. The selected PE-SNEDDS was adsorbed on the porous material surface i.e., avicel PH-101 to develop S-PE-SNEDDS. The developed S-PE-SNEDDS was further evaluated for in-vitro characteristics followed by an antimicrobial activity, pharmacokinetic study, and pharmacodynamic study.

## 2. Materials and Methods

### 2.1. Materials

Beijing Mesochem Technology Co. Pvt. Ltd. (Beijing, China) provided the Piperine (98.21% purity). Eucalyptus oil, Almond oil, Sunflower oil, and Ethyl oleate were procured from SD-fine chemicals (Mumbai, India). Labrafac WL, Caprol PGE-860, Capmul MCM, Labrafil M1944 CS, and Transcutol HP were procured from the Gattefosse Mumbai, India. Glyceryl monolinoleate (GML) Cremophor RH 40, Solutol HS 15, and Span 20 were obtained from the BASF India Ltd. (Bandra East, Mumbai, India). Polyethylene glycol-400 (PEG-400), PEG200, propylene glycol (PG), and hydrochloric acid were purchased from Acros organic (Mumbai, India). Poloxamer188, poloxamer 127, and avicel PH101 were obtained from Sigma Aldrich (St Louis, MO, USA).

### 2.2. Methods

#### 2.2.1. Screening of Formulation Materials

Solubility of PE in individual components (oil, surfactant, and co-surfactant) was performed to select the final component to prepare the SNEDDS. The appropriate quantity (1 mL) of oils (GML, labrafac WL, eucalyptus oil, almond oil, sunflower oil, ethyl oleate, caprol PGE-860, capmul MCM, and Labrafil M1944 CS), surfactants (poloxamer 188, poloxamer 127, cremophor RH 40, solutol HS 15 and span 20) and co-surfactants (PEG400, PEG200, PG, and Transcutol HP) were taken into glass vials. An excess of PE was added to each vial and then vortexed for mixing. Each mixture was shaken for 72 h and then centrifuged at 6000 rpm for 30 min to separate the supernatant. The concentration of PE in each sample was evaluated after proper dilution by using a UV-visible spectrophotometer (UV-1800, Shimadzu, Kyoto, Japan) at 340 nm.

#### 2.2.2. Construction of Pseudo Ternary Phase Diagram

The ternary phase diagram was built between the chosen components (oil, surfactant, and co-surfactant) of SNEDDS using the aqueous phase titration method for estimation of self-emulsification efficiency [19]. The surfactant and co-surfactant (Poloxamer188, Transcutol HP, Smix) were mixed properly with oil (GML) in different compositions (3:7, 4:6, 5:5, 6:4, 7:3, 8:2, 9:1) in a glass beaker and titrated with mili Q-water with continuous magnetic stirring. The mixture was observed visually for turbidity and any phase changes. The self-nanoemulsifying region was determined by plotting the phase diagram in triangular co-ordinate software. Further, based on the selected emulsifying region, the low (−), medium (0), and high (+) level of oil, surfactant, and co-surfactant were selected for optimization using Box-Behnken (Table 1) [20].

### 2.3. Optimization

The Box-Behnken design (Design Expert software, Stat-Ease, Minneapolis, MN, USA) was used to analyze the influence of formulation variables (individual, interaction, or quadratic effect) over the responses. It gives a lesser number of formulation compositions with the used components. The optimization was done by evaluating globule size (Y_1_, nm), transmittant (Y_2_, %), and emulsification time (Y_3_, seconds) by employing three independent factors: oil (A), surfactant (B), and co-surfactant (C) at three levels (Table 1). The design showed 17 formulation compositions with five common formulae to assess error. The results were evaluated on different models: linear, 2F1, and quadratic, to assess the effect of formulation factors. ANOVA, polynomial equation, and response surface plot were also used to evaluate the effect of independent factors [20].

### 2.4. Development of PE Loaded SNEDDS

The optimized SNEDDS (F1-F17) compositions were obtained from Box Behnken design using the designated components: oil (A), surfactant (B), and co-surfactant (C) as shown in Table 1. The appropriate quantity of selected components was mixed into a glass vial to form a uniform homogeneous mixture. The calculated amount of PE (20 mg) was added in the SNEDDS (F1-F17) followed by vortexing to obtain a homogeneous mixture of PE-SNEDDS [21,22]. PE-SNEDDS was preserved at ambient temperature for further study.

### 2.5. Characterization

#### 2.5.1. Globule Characterization

Zeta sizer ((Malvern zeta sizer, Malvern, UK) was used to determine the globule size, PDI, and zeta potential of PE-SNEDDS with appropriate dilution. The morphological investigation of optimized PE-SNEDDS (OF3) was performed by a transmission electron microscope (JEM1011, JEOL, Tokyo, Japan).

#### 2.5.2. Self-Nano Emulsification Time

The self-emulsification time was examined with the help of the paddle type of USP dissolution apparatus (Sotex AG, Aesch, Switzerland). The distilled water (500 mL) was filled into a dissolution basket and the temperature was fixed at 37 ± 0.5 °C. The developed PE-SNEDDS was added into water drop by drop with continuous stirring at 100 rpm. The emulsification time of each formulation was observed in triplicate and the time was noted.

#### 2.5.3. Percentage Transmittance

The percentage transmittance was examined by using the UV-visible spectrophotometer at 638 nm. PE-SNEDDS was reconstituted with water (100 times), and the transmittance was observed in triplicate.

### 2.6. Evaluation of Optimized PE-SNEDDS (OF3)

The optimized PE-SNEDDS (OF3) was centrifuged (Hettich, Tuttlingen, Germany) at 6000 rpm for 20 min after dilution with water (1:100). The sign of instability like phase separation, phase inversion, and creaming were inspected visually. The optimized PE-SNEDDS (OF3) was kept at a temperature between 4–40 °C for 48 h for three cycles and assessed for any instability. The freeze-thaw study was performed by taking the reconstituted sample and kept at −20 to 25 °C (48 h) for three cycles. Then, the formulation was centrifuged and observed for any instability (phase separation, creaming). The viscosity was assessed by Brookfield viscometer (DVT2 viscometer, Middleboro, MA, USA) for optimized PE-SNEDDS (OF3) formulation. The viscosity (cp) was measured at 40 rpm using 31S spindle size. The refractive index was measured by a refractometer (Abbes-refractometer, Hamburg, Germany) at 25 ± 2 °C.

### 2.7. Drug Release Study

The release of PE form optimized PE-SNEDDS (OF3) and pure PE-dispersion was performed by using basket-type USP dissolution type (Sotex AG, Aesch, Switzerland). The release media (0.1*N* HCl) was prepared and filled into a dissolution basket, and temperature was maintained at 37 ± 0.5 °C. The optimized PE-SNEDDS (OF3 containing ~5 mg PE) was filled into a pretreated dialysis bag and both the ends were tightly tied. The dialysis bag was tied with a paddle and rotates at 50 rpm. At a definite interval, 5 mL of the released sample was withdrawn from the basket and the same volume was replaced with fresh media. The concentration of PE was investigated by a UV-visible spectrophotometer at 340 nm. The dissolution of pure PE-dispersion was also determined by the same procedure for comparative study.

### 2.8. Permeation Study

The permeation study was performed using the rat intestine. The rats were kept in a fasted state for 12 h before the start of study. The fresh intestine was collected immediately after the sacrifice and the intestine was collected, washed with ringer solution, and stored. The optimized PE-SNEDDS (OF3) and pure PE-dispersion were filled (equivalent to 2 mg PE) and tied from both ends. The intestine was immersed into a ringer solution (100 mL) as release media with continuous stirring. Two milliliter of an aliquot was taken at a predetermined time interval, and the same volume was added to the beaker. The amount of PE permeated for each sample was measured by the previously reported HPLC method [23]. The graph was plotted between the amount of drug permeated vs. time, and the apparent permeability coefficient was calculated by the given mathematical Equation (1):

(1) $$\mathrm{Apparent}\;\mathrm{permeability}=\frac{Flux}{Area\times Initial\;drug\;concentration}\;\;$$

### 2.9. Formulation of Solid PE-SNEDDS (S-PE-SNEDDS)

The optimized PE-SNEDDS (OF3) transformed into a solid form by adsorbing on a suitable adsorbent i.e., avicel PH-101. The appropriate quantity of adsorbent was taken in a porcelain dish and optimized PE-SNEDDS was added dropwise with proper mixing. The formulation was sieved with a 120 µm size sieve for fine and uniform size and preserved under room condition for further evaluation [24].

### 2.10. Evaluation of S-PE-SNEDDS

#### 2.10.1. Globule Size, PDI, and Zeta Potential

All three parameters were measured as per the procedure given in the globule characterization of SNEDDS section.

#### 2.10.2. Morphological Study

The surface structure of pure PE, avicel PH-101, and S-PE-SNEDDS (SOF3) were analyzed by scanning electron microscope (IRMC- INSPECT S50 Tokyo, Japan). The samples were held with double-sided tape and subsequently coated with gold. The thin layer of the sample was analyzed at an accelerated voltage of 15 kV to capture the image.

#### 2.10.3. Thermal Analysis

The thermal analysis of pure PE, avicel PH101, and S-PE-SNEDDS (SOF3) were evaluated to check the changes in the PE characteristics. The samples were kept in an aluminum pan and scanned under nitrogen inert conditions at 25–450 °C with the help of a differential scanning calorimeter (DSC, Mettler, Toledo, Columbus OH 43240, USA). 

#### 2.10.4. Drug Content

The drug content of optimized S-PE-SNEDDS (SOF3) was analyzed to check the amount of drug present in the final formulation. The % content of PE was determined by spectrophotometer, and PE content was calculated by the below Equation (2):

(2) $$\%\;\mathrm{Drug}\;\mathrm{content}\;=\frac{Final\;drug\;concentration}{Inital\;concentration\;}\times 100\;\;$$

#### 2.10.5. Drug Release Study

The dissolution was carried out in 0.1 *N* HCL (pH 1.2) as a release medium. The release media (900 mL) was filled into dissolution apparatus (USP-dissolution apparatus-I, Sotex AG, Aesch, Switzerland), and the temperature was maintained at 37 ± 0.5 °C [25]. The required amount of S-PE-SNEDDS (SOF3) was filled into a hard gelatin capsule (0 size). The capsule was placed in a dissolution basket and rotated at 75 rpm. At specific time intervals, an aliquot (5 mL) was collected and simultaneously replaced with the same volume of fresh release media. The amount of PE released at each time was examined by a UV-visible spectrophotometer at 340 nm. The release data of S-PE-SNEDDS (SOF3) was fitted into various release kinetic models to find out the best-fit model. 

#### 2.10.6. In-Vitro Antioxidant Study

Free radical scavenging activity (RSA) of five different concentrations of PE formulations was done by using the standard DPPH method [26]. Five different concentrations of pure PE and S-PE-SNEDDS (SOF3) were prepared separately in ethanol. DPPH solution (0.02%, 125 µL, violet color) was prepared in ethanol and mixed with each sample (500 µL) of S-PE-SNEDDS (SOF3) and pure PE. The reaction mixture was agitated for 1 h under the dark condition to complete the reaction. At the endpoint, a chemical reaction between the DPPH solution (originally violet color) and the antioxidant molecule gives a colorless solution. The samples were examined at 517 nm with the help of a UV spectrophotometer using ethanolic DPPH solution as a blank [27]. The antioxidant activity of each sample was examined in triplicate by the application of Equation (3):

(3) $$\mathrm{AA}\;\left(\%\right)=\frac{Control\;sample\;absorbance-Test\;sample\;absorbance}{Control\;sample\;absorbance}\times 100\;\;$$

#### 2.10.7. Anti-Microbial Study

Antimicrobial activity of S-PE-SNEDDS (SOF3) and pure PE was conducted on *Staphylococcus aureus* (Gram’s positive) and *Pseudomonas aeruginosa* (Gram’s negative) by diffusion technique [28,29]. The bacterial strains were sub-cultured on nutrient broth under specified conditions and serially diluted to evaluate the different samples. The nutrient agar Petri plates were prepared (25 mL) under aseptic conditions and each bacterial strain was spiked (500 μL, 1 × 10^6^ CFU/mL concentration) into sterile Petri dishes. The samples were incubated for 1 h for solidification. Three wells (6 mm diameter) were created in each Petri plate by the application of a sterile borer. Each sample (around 1 mL) was transferred to the wells and incubated for 24 h at 37 ± 1 °C. Each experiment was performed three times against each strain to calculate the mean zone of inhibition. The well with bacterial growth (incubated with sterile water) was taken as a control. 

### 2.11. In-Vivo Study

The in-vivo study was done on albino Wistar rats with the approved protocol (number 18-5-42). The approval for the protocol was given by in Jouf University institutional animal ethical committee, Al-Jouf, KSA. The albino rats (200–250 gm) were obtained from the institutional animal house and kept under appropriate environmental conditions (25 ± 2 °C) with 12 h day/night cycle. The rats were supplied with a free assessment of standard normal diet and water supplied ad libitum. 

#### 2.11.1. Pharmacodynamic Study

The rats were divided into four different groups. Group 1 was designated as normal control, group 2 as hypertension control, group 3 as pure PE-dispersion-treated, and group 4 as S-PE-SNEDDS (SOF3)-treated group. The rats were trained to stay in a restrainer every day and before the start of the study. The rats were acclimatized then hypertension was induced by the subcutaneous administration of methylprednisolone (MP) (Depo-Medrol, 40 mg/mL) for 1 week to groups 2, 3, and 4. The BP instrument (LE 5002 Pressure Meter, Harvard apparatus, MA, USA) was used to measure the blood pressure (systolic and diastolic) before and after induction of hypertension. The pure PE-dispersion and S-PE-SNEDDS (SOF3) (20 mg/kg) were administered orally in group 3 and group 4 [30]. The systolic and diastolic BP were measured by placing the restrainer carrying the rats, and the tail was cuffed with the sensor. Then, the thermosensitive key of the instrument was pressed and BP was recorded. 

#### 2.11.2. Pharmacokinetic Study

The animals were divided into two groups each containing six rats. The animals in Group I were used for pure PE and group II for S-PE-SNEDDS (SOF3). The rats were kept in 12 h fasting condition before the drug administration. The rats were cannulated with a Smiths Medical^TM^ Portex^TM^ polyethylene catheter (Fisher Scientific Ltd., Loughborough LE11 5RG, UK) in the left jugular vein for the collection of blood. The pure PE dispersion and S-PE-SNEDDS (SOF3) (20 mg/kg) were orally administered to each rat [30]. The blood sample from each rat were collected at 0.25, 0.5, 1, 2, 3, 6, and 12 h and placed into an Eppendorf tube containing EDTA. The blood sample was centrifuged at 4000 rpm for 15 min, and the plasma sample was separated by micropipette and homogenized with formic acid (0.2%) and ethyl acetate to extract PE [23,31]. Both samples were dried under vacuum conditions with the help of a vacuum dryer. Finally, the extract was reconstituted with mobile phase and filtered by cellulose membrane (0.45 µm). The sample was injected (20 µL) into the HPLC column for the analysis of PE. The analysis was conducted at room temperature with a flow rate of 1 mL/min, and the duration of the run was 10 min. The plasma PE concentration vs. time graph was plotted for the determination of various pharmacokinetic parameters (C_max_, T_max_, AUC_0–t_, AUC_0–∞,_ elimination rate constant, and half-life) by considering the non-compartment model.

#### 2.11.3. Histological Study

From the sacrificed animals, the intestinal mucosa was separated, cleaned, and transferred to a formalin solution (10%). The mucosa was treated with alcohol followed by xylene and finally fixed in the paraffin wax. Thin mucosal sections (4 µm) were cut by microtome and stained with hematoxylin-eosin (H&E) dye. The histology of each section was examined with the help of a light microscope (BX51, Olympus, Tokyo, Japan). 

### 2.12. Stability Study 

The stability study of optimized S-PE-SNEDDS (SOF3) was performed at 40 ± 2 °C/75 ± 5% RH for 6 months (“European Medicines Agency”, 2020). The formulation was packed in a glass vial and kept in a stability chamber (Hicon, Mumbai, India). The samples were withdrawn at a predetermined time, and the globule size, % transmittant, and emulsification time were measured. For shelf-life calculation, the optimized S-PE-SNEDDS (SOF3) was stored at different temperatures (30 ± 2 °C, 40 ± 2 °C, 50 ± 2 °C, and 60 ± 2 °C) for 6 months. The samples were withdrawn at predetermined time points (0, 1, 2, 3, and 6 months) and drug content was measured. The degradation rate constant (*K*) was determined with the help of Equation (4). Arrhenius plot was constructed between log *K* and reciprocals of various temperatures, and *K* value at 25 °C was determined. The shelf-life at (t90) by Equation (5):

(4) $$\mathrm{Slope}=\frac{-K}{2.303}\;$$

(5) $$\mathrm{Shelf}\;\mathrm{Life}\;\left(t90\right)=\frac{0.1052}{K25℃}\;$$

### 2.13. Statistical Analysis

Graph Prism Pad was applied for statistical analysis. For comparison, *p* < 0.05 was taken to detect the significant difference. One-way ANOVA and Tukey Karman statistical analysis were applied for comparison. All the data were presented as mean ± SD. 

## 3. Results and Discussion

### 3.1. Screening of Formulation Materials

**Figure 1:**
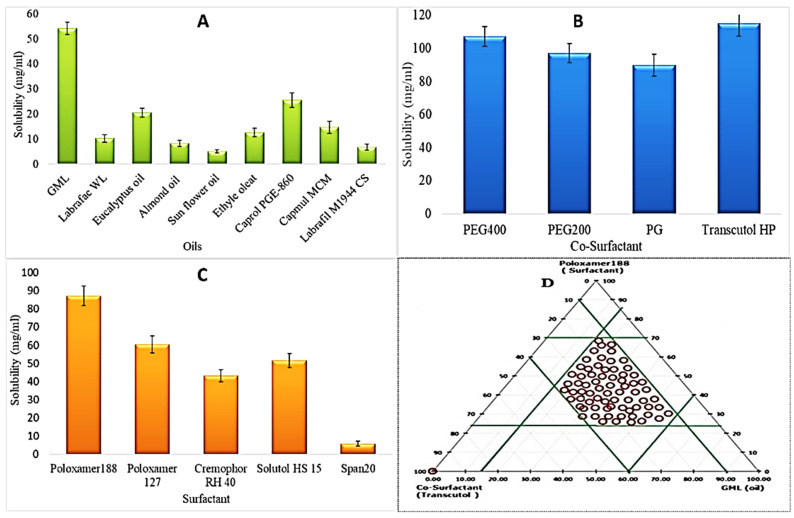
(**A**–**D**): Showing the solubility behavior of PE in various vehicles like surfactant (**A**), co-surfactants (**B**), and oils (**C**). Ternary phase diagram of PE SNEDDS formulation (**D**). Red covered area in ternary phase diagram represents the experimental domains for BBD. Values are given as mean ± SD (*n* = 3).

The oil, surfactant, and co-surfactant were screened based on the maximum solubility of PE in the respective components, and data are given in Figure 1. The high solubility of the drug in oil is good for self-emulsification and solubilization. Moreover, high drug lipid solubility also assists in intestinal drug absorption through the lymphatic pathway [32]. The solubility order of PE in oil is Glyceryl monolinoleate (GML) > Caprol PGE-860 > Eucalyptus oil > Capmul MCM > Ethyl oleate > Labrafac WL > Almond oil > Labrafil M1944 CS > Sunflower oil (Figure 1A). PE exhibited the maximum solubility in GML (54.23 ± 2.5 mg/mL). The order of PE in various surfactants are Poloxamer188 > Poloxamer 127 > Solutol HS 15 > Cremophore RH 40 > span20 (Figure 1B). The maximum solubility of PE was found in Poloxamer188 (87.23 ± 5.34 mg/mL). The poloxamer 188 has an HLB value of 29, and it is a hydrophilic surfactant and supports the quick formation of *o*/*w* droplets with high emulsification efficiency. The solubility order of PE in co-surfactants is as follows Transcutol HP > PEG400 > PEG200 > PG (Figure 1C). PE has shown maximum solubility in Transcutol HP. Based on the solubility study, GML, Poloxamer 188, and Transcutol HP were screened as oil, surfactant, and co-surfactant, respectively, for the design of PE-loaded SNEDDS (PE-SNEDDS). 

### 3.2. Pseudo Ternary Phase Diagram

A ternary phase diagram was constructed by taking the components that were finalized in the solubility study. Here, GML, poloxamer 188, and Transcutol HP were taken as oil, surfactant, and co-surfactant, respectively. The purpose of the phase diagram was to dispose of the sufficient self-emulsifying region. The feasibility of nanoemulsion formation depends upon the extreme values of the excipients. Therefore, the extreme and middle levels of the independent factors viz oil, surfactant, and co-surfactant, were designated for further processing [33]. In Figure 1D, the red zone exhibiting the efficient self-nanoemulsifying region where all the desired characters, namely clarity of the solution without phase separation and spontaneous emulsion formation, were perceived.

### 3.3. Optimization 

The SNEDDS was developed by the selected excipients, and a further three-factor at three-level Box-Behnken design was employed for optimization. The prepared formulations were evaluated for different parameters, and their results are shown in Table 2. The polynomial equation of each response is given below

Globule size (Y_1_) = +103.00 + 56.81A − 29.89B − 20.45C − 12.51 AB + 0.26AC + 6.25BC − 4.76A^2^ + 4.25 B^2^ + 4.00C^2^ (6)

Percentage Transmittance (Y_2_) = +97.79 −3.43 A + 3.97 B + 3.00 C + 3.55 AB − 1.28 AC − 0.81 BC 2.39A^2^ − 1.24 B^2^ − 2.19C^2^ (7)

Emulsification time (Y_3_) + 62.20 + 29.42A − 18.87B − 22.26C − 12.50AB − 7.57AC + 13.25BC + 22.56 A^2^ − 2.26 B^2^ − 8.19C^2^ (8)

Equations (6)–(8) explained the effect of formulation variables (A—oil, B—surfactant, C—co-surfactant concentration) on the globule size (Y_1_), percentage transmittant (Y_2_), and emulsification time (Y_3_), individually (A, B, C) and in combination (AB, BC, and AC), respectively. The factors A, B, C, AB, BC, A^2^, B^2^, and C^2^ are the significant (*p* < 0.05) model terms and significantly affect the globule size (Y_1_). The combined factors (AC) represented a nonsignificant (*p* < 0.05) effect on globule size. On the other side, all model terms i.e., A, B, C, AB, AC, BC, A^2^, B^2^, and C^2^ significantly affect the percentage transmittant (Y_2_) and emulsification time (Y_3_). The positive and negative signs indicate the positive and negative responses (Y_1_, Y_2_, Y_3_). All the responses (Y_1_, Y_2_, Y_3_) showed the quadratic model and were found as the best fit model due to the maximum regression coefficient (R^2^) value compared to that of other models (linear and 2nd order) (Table 3). The effectiveness of the quadratic model for each response was confirmed by ANOVA. The lack of fit was found to be insignificant (*p* > 0.05) for each response, indicating a nonsignificant deviation between the actual and predicted value. The adequate precession of the fitted model was found to be >4, showing the model is well fitted and desirable. The 3D plot of each response was constructed to explain the effect of individual and interaction effect of variables over the responses.

#### 3.3.1. Effect of Variables over Globule Size (Y_1_)

**Figure 2:**
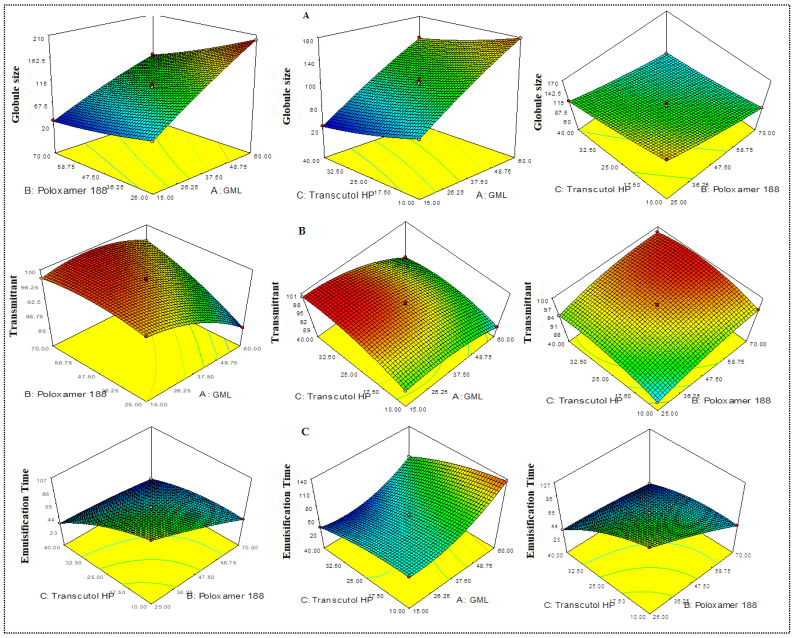
3D response surface plot showing the effect of independent variables on the globule size (**A**), transmittance (%) (**B**), and emulsification time (**C**).

All PE-SNEDDS showed globule size in the range of 25.16 ± 6.17 nm (F7)–200.34 ± 4.23 nm (F2). The oil concentration (A) showed a positive effect on globule size (Y_1_). The increase in oil concentration of the globule size (F2) also increases due to insufficient emulsification at constant surfactant and co-surfactant [34,35]. On the contrary, on increasing the surfactant (B) and co-surfactant concentration (C), the globule size (Y_1_) decreased due to the greater availability of surfactant and co-surfactant at the oil/water interface. It diminishes the interfacial tension and delivers stability to the system/globules. These results agreed with the previously published work [20]. When both oil and surfactant concentration increase, the globule size also increases due to the liquid crystal as well as an increase in viscosity of the system (F9). The 3D response plot (Figure 2A) for globule size expressed the combined effects of the independent variables over the response. 

#### 3.3.2. Effect of Variables over Percentage Transmittant (Y_2_)

The percentage transmittant of all formulations was analyzed, and data are represented in Table 2. % Transmittant indicated the clearness or cloudiness of PE-SNEDDS. The % transmittant of all PE-SNEDDS was found in the range of 88.54 ± 1.27 (F9)–99.89 ± 2.46% (F7). The oil concentration (A) exhibited a negative influence. The increase in oil concentration % transmittant reduced due to insufficient emulsification (F2) [28,29]. With the increase in the amount of surfactant and co-surfactant, the % transmittant was increased due to the greater availability of these components at the oil and water interface. These results are in line with the previously published work [20]. A 3D plot of percentage transmittant is depicted in Figure 2B, and it showed the individual as well as combined effects of independent variables over the response. 

#### 3.3.3. Effect of Independent Variables over Emulsification Time (Y_3_)

The results of this parameter depicted in Table 2. The emulsification time of PE-SNEDDS was found in the range of 23 ± 3 (F12)–144 ± 3 s (F2). The polynomial equation of emulsification time exhibited an increase in oil concentration (A), showing a positive effect. With the increase in oil concentration, the emulsification time increases because of insufficient emulsification [34,35]. On the other hand, on increasing the surfactant and co-surfactant concentration, the emulsification time was also increased due to the availability of surfactant and co-surfactant at the interface of oil and water, which diminishes the interfacial tension. These results are in agreement with the previously published work [20,36]. The 3D plot expressed the combined effects of the independent variables over the response (Figure 2C).

### 3.4. Point prediction

Further, the formulation was optimized by the point prediction method. The optimum composition with the actual and predicted result was given in Table 4. The optimized composition was found with oil (GML 25%), surfactant (poloxamer 188, 46.28%), and co-surfactant (Transcutol HP, 25%). The predicted value of optimized formulation (PE-SNEDDS, OF3) has a globule size of 71.21 nm, transmittant of 98.87%, and emulsification time of 54 sec. The experimental value of the optimized formulation (PE-SNEDDS, OF3) shows the globule size 70.34 ± 3.27 nm, transmittant 99.02 ± 2.02%, and emulsification time 53 ± 2 s.

### 3.5. SNEDDS Characterization 

**Figure 3:**
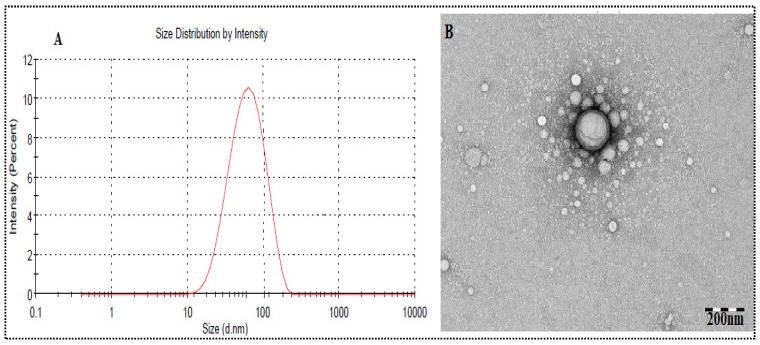
Showing: (**A**) Globule size and (**B**) TEM image of optimized PE-SNEDDS (OF3).

The globule size of the optimized formulation (OF3) was observed at 70.34 ± 3.27 nm (Figure 3A). The PDI is 0.351 (<0.5), which indicated the homogeneous distribution of globules. The zeta potential was found to be −27.34 mV, indicating the physical stability of SNEDDS without aggregation of the globule [37]. The morphology of optimized PE-SNEDDS (OF3) was determined by TEM and found to be spherical (Figure 3B). 

### 3.6. Stability Study

SNEDDS can dissolve the drug in the form of nanoemulsion after emulsification. It comes in contact with aqueous media without any sign of physical instability i.e., cracking, creaming, and phase separation [38]. The optimized PE-SNEDDS formulation (OF3) was tested with different stressed conditions, viz. centrifugation test, heating, cooling cycle, and freeze-thaw cycle. The formulation did not exhibit any physical instability sign like cracking, creaming, phase separation, and turbidity, therefore, it passes the test. 

### 3.7. Viscosity and Refractive Index Measurement 

The optimized PE-SNEDDS formulation (OF3) showing the viscosity and refractive index of 95.76 ± 1.65 cps and 1.314 ± 0.03. The values were found closer to the refractive index of water (1.33).

### 3.8. Drug Release 

**Figure 4:**
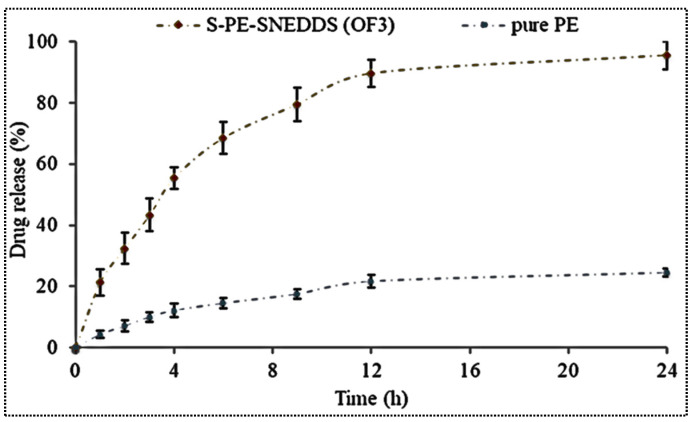
In-vitro release profile of SNEDDS and pure PE dispersion. Values are given as mean ± SD (*n* = 3).

The % release from PE-SNEDDS (OF3) and pure PE-dispersion was calculated to show the difference in release profile (Figure 4). The formulation OF3 showed an initial burst release (26.33 ± 4.23% in 2 h) and later sustained drug release (91.01 ± 3.21% in 24 h). The initial fast release may be due to the presence of free PE at the surface of SNEDDS. Later, the prolonged PE release was found due to the release of entrapped PE in SNEDDS. Moreover, the pure PE-dispersion displayed a significantly poorer release (*p* < 0.05) than SNEDDS due to the low aqueous solubility.

### 3.9. Permeation Study

**Figure 5:**
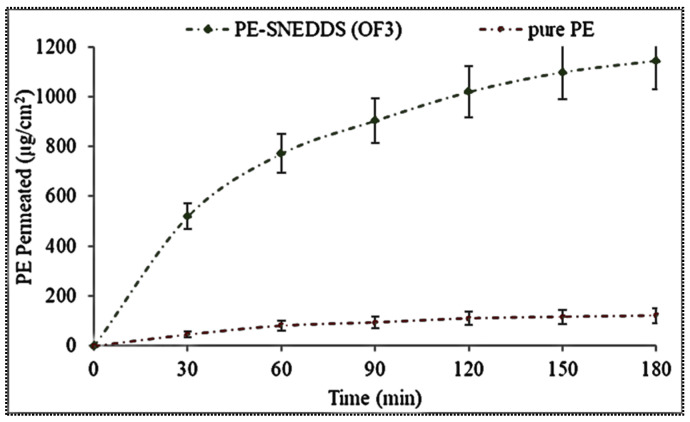
Ex vivo permeation profile of PE-SNEDDS (OF3) and pure PE dispersion. Values are given as mean ± SD (*n* = 3).

The fresh rat intestine was employed for the ex-vivo permeation study, and the result of this study is depicted in Figure 5. The PE-SNEDDS (OF3) exhibited a significantly (*p* < 0.05) higher amount of PE permeation (1145.23 ± 94.5 µg) than the PE-dispersion (120.43 ± 13.87 µg) in 3 h of study. The higher permeation is due to the greater solubilization of PE in SNEDDS. The flux of OF3 was found to be 5.87 µg/min/cm^2^, which is 9.32-fold more than PE dispersion (0.6298 µg/min/cm^2^). The apparent permeability of OF3 was found to be significantly (*p* < 0.05) higher (19.57 × 10^−4^ cm/min) than that of pure PE dispersion (2.1 × 10^−4^ cm/min).

### 3.10. Development of Solid PE-SNEDDS

A biocompatible porous material (avicel PH-101) was used for the successful transformation of optimized PE-SNEDDS (OF3) into S-PE-SNEDDS (SOF3) (Table 5). 

### 3.11. Characterization 

#### 3.11.1. Globule Size, PDI, Zeta potential

The S-PE-SNEDDS (SOF3) powder was suitably diluted with deionized water for the analysis of globule size, PDI, and zeta potential. The globule size, PDI, and zeta potential were found to be 73.56 ± 3.54 nm, 0.352 ± 0.023, and −28.12 ± 2.54 mV. These values are slightly greater than OF3 due to reconstitution.

#### 3.11.2. Scanning Electron Microscopy (SEM)

**Figure 6:**
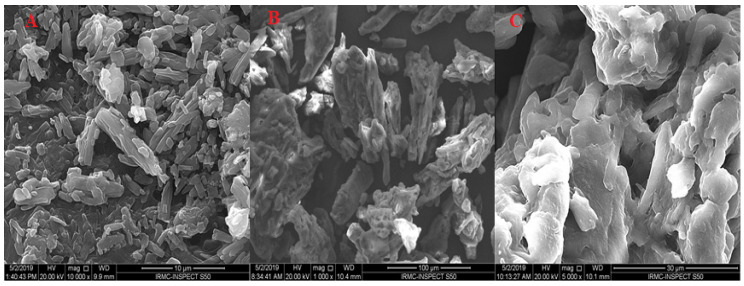
SEM image of (**A**) pure PE, (**B**) Avicel PH101, and (**C**) S-PE-SNEDDS (SOF3).

Figure 6 displays an image of pure PE, avicel PH-101, and S-PE-SNEDDS (SOF3). The pure PE displayed an asymmetric-shaped crystal with dissimilar sizes (Figure 6A). Moreover, avicel PH-101 exhibited porous rough particles (Figure 6B). No oil droplets were found in the SEM image of S-PE-SNEDDS (SOF3). It showed porous unequal-shaped particles. It confirms that S-PE-SNEDDS completely adsorbed on the surface of the adsorbent (Figure 6C).

#### 3.11.3. Thermal Analysis

**Figure 7:**
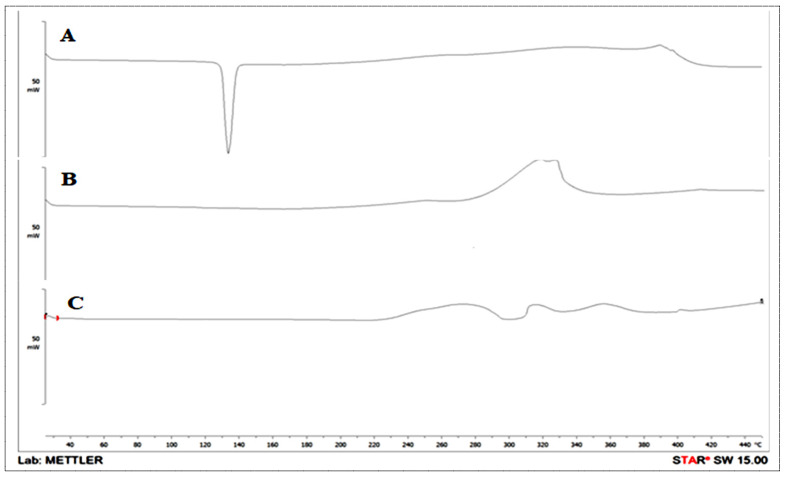
DSC thermogram of (**A**) pure PE, (**B**) Avicel PH-101, and (**C**) S-PE-SNEDDS (SOF3).

The pure PE, avicel PH-101, and S-PE-SNEDDS (SOF3) were evaluated for thermal analysis by differential scanning calorimetry and thermogram depicted in Figure 7. The pure PE thermogram demonstrated a sharp intense peak at 135.4 °C, which is closer to its melting point (Figure 7A). Avicel exhibited a peak at 300 °C (Figure 7B), which is closer to the reported value. However, no characteristic peak of PE was found in S-PE-SNEDDS (SOF3) thermogram (Figure 7C), showing complete solubilization of PE in the oil and surfactant [38].

#### 3.11.4. Drug Release Study

**Figure 8:**
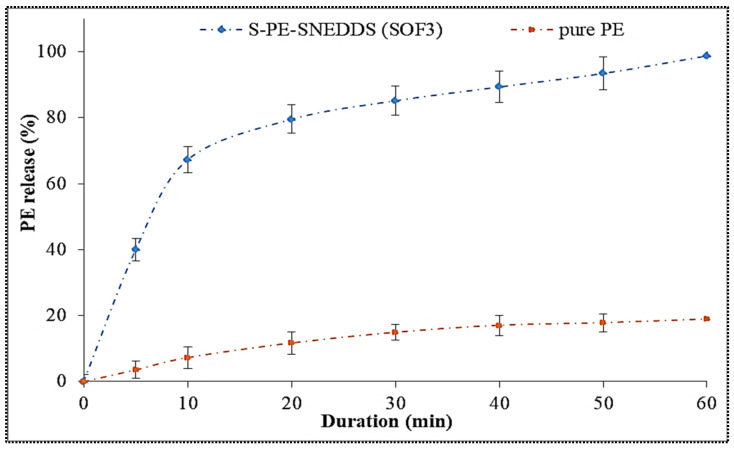
Comparative dissolution study of S-PE-SNEDDS (SOF3) and pure PE dispersion. Values are given as mean ± SD (*n* = 3).

Figure 8 displays the in-vitro release profile of S-PE-SNEDDS (SOF3) and pure PE. The S-PE-SNEDDS (SOF3) exhibited 46.32 ± 2.31% PE release in the initial 10 min and 97.87 ± 4.89% in 60 min. However, the pure PE showed only 18.87 ± 2.65% in 60 min due to the low aqueous solubility. There was a highly significant (*p* ˂ 0.001) difference in the release found between both samples. S-PE-SNEDDS showed fast PE release due to the high self-emulsification property (less surface energy) between the oil and release media. The presence of surfactant and co-surfactant also helps to reduce the interfacial tension and promotes the drug release. The other reasons for the improvement of PE release are the small globule size, quick emulsification, large porous surface area, and loss of the crystallinity of PE in S-PE-SNEDDS (SOF3). The release profile was fitted to different kinetic models and the results depicted in Table 6. Among all applied models, zero-order model was selected as the best-fit model due to the highest regression coefficient value (R^2^ = 0.9631). The mechanism of drug release was found to be the Fickian diffusion mechanism (*n* = 0.2096).

#### 3.11.5. Antioxidant Study

**Figure 9:**
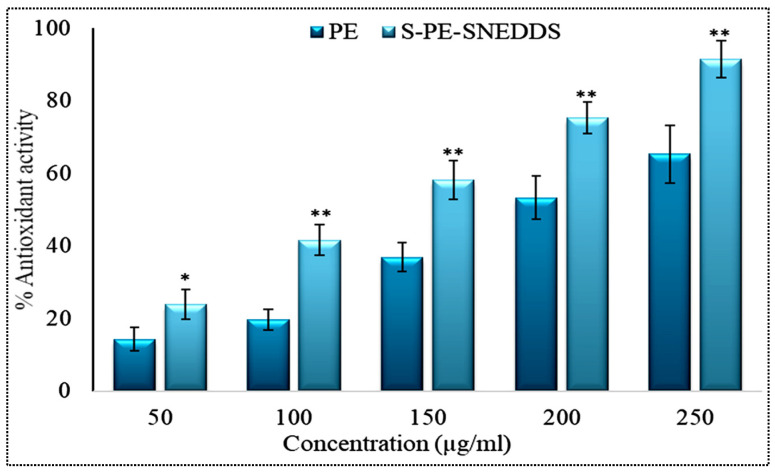
Comparative antioxidant profile of pure PE dispersion and optimized S-PE-SNEDDS formulation. Values are given as mean ± SD (*n* = 3), * significantly different at *p* < 0.05 and ** *p* < 0.01.

DPPH solution is mixed with a substrate that has antioxidant activity; its original violet color is lost due to the formation of a reduced form. It acts by accepting the electron/hydrogen from the substrate [39]. A comparative antioxidant activity was performed between SOF3 and pure PE dispersion as shown in Figure 9. From the figure, it is concluded that the scavenging activity of both samples was increased with increasing the concentration. The highest scavenging activity of the S-PE-SNEDDS (SOF3) was found to be 91.48 ± 3.23% at 250 µg/mL concentration, which was significantly (*p* < 0.001) higher than pure PE dispersion (69.37 ± 2.65%). A significant (* *p* ˂ 0.05, ** *p* ˂ 0.001) difference in the antioxidant activity was observed between SOF3 and pure PE at each concentration. 

#### 3.11.6. Anti-Microbial Activity

The potential of the developed formulation was examined in the terms of ZOI. ZOI for S-PE-SNEDDS (SOF3) was found to be 18.97 ± 1.39 mm and 17.82 ± 0.95 mm against *S. aureus* and *P. aeruginosa,* respectively. These values were significantly higher than pure PE dispersion. The pure PE dispersion showed ZOI of 15.73 ± 0.87 mm and 13.56 ± 1.28 mm. The above-stated results might be due to the higher PE solubility and nanosized S-PE-SNEDDS (SOF3). At higher solubility, more drug internalization takes place within the microorganism cell, causing the disruption of the cytoplasmic membrane (infiltrating out the cytoplasmic content and cell wall fragmentation) and the cell wall [40,41]. Therefore, PE is exhibiting slightly more antimicrobial activity. These findings are in line with the previous study as they reported that PE acts as a potent antibacterial agent and with the good inhibiting property of efflux pump of *S. aureus* [42].

### 3.12. In Vivo Study

#### 3.12.1. Pharmacodynamic Activity

**Figure 10:**
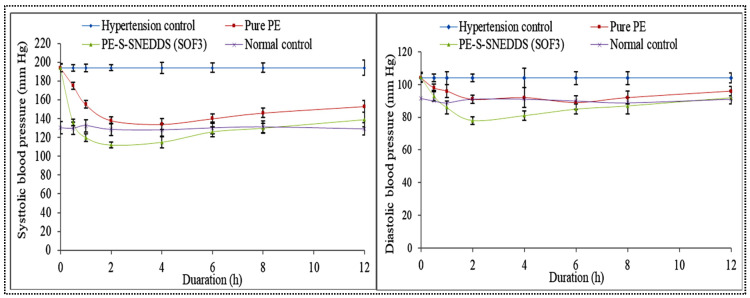
Antihypertensive potential (left) Systolic BP and (right) diastolic BP of hypertension group, pure PE, S-PE-SNEDDS (SOF3), and normal group rats. Values are given as mean ± SD (*n* = 6).

The antihypertensive activity of pure PE and S-PE-SNEDDS (SOF3) was done in MP-induced hypertensive Wistar albino rats, and the result is expressed in Figure 10 (left, right). The hypertension was successfully induced in normal rats after the administration of MP. In the disease control group, the systolic and diastolic BP was found to be 194.32 ± 4.45 mmHg and 104 ± 2.98 mmHg, respectively. It remained constant throughout the study duration (12 h). The normal control group showed systolic and diastolic BP 130.54 ± 3.65 mmHg and 91.45 ± 2.19 mmHg, respectively. The BP was more significantly reduced (*p* < 0.05) after oral administration of pure PE and S-PE-SNEDDS (SOF3) than the disease control group. The pure PE dispersion-treated group displayed a decline of systolic BP 160.21 ± 3.54 in 0.5 h and 135.43 ± 3.24 mmHg in 2 h and then gradually increased up to 12 h. There was also a significant (*p* < 0.05) reduction in diastolic BP i.e., 98.43 ± 3.12 in 0.5 h and 92.32 ± 2.26 mmHg in 2 h and then BP increased. However, S-PE-SNEDDS (SOF3)-treated group showed reduced systolic BP 136.36 ± 3.54 mmHg and 113.56 ± 3.54 at 0.5 h and 1 h. It also reduced the diastolic BP to 93.12 ± 2.33 and 85.05 ± 2.34 mmHg at the same time, respectively. It was observed that S-PE-SNEDDS (SOF3) exhibited a significantly (*p* < 0.05) higher reduction in systolic and diastolic BP as compared to pure PE-dispersion. It is due to quick self-emulsification in the gastric region that forms self-nanoemulsion, which is dissolved and absorbed through the lymphatic pathway and attributes to the quick onset of action [25].

#### 3.12.2. Pharmacokinetic Study

**Figure 11:**
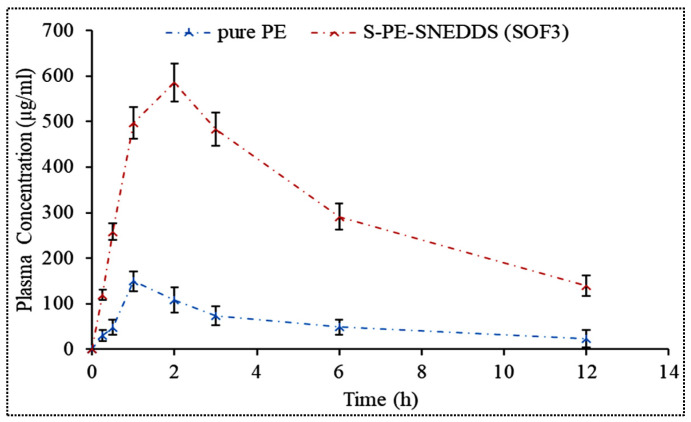
Plasma concentration time profile of S-PE-SNEDDS-opt and pure PE. Values are given as mean ± SD (*n* = 6).

The pharmacokinetic study was performed on Wistar rats after oral administration of S-PE-SNEDDS (SOF3) and pure PE dispersion (Figure 11). The pharmacokinetic parameters i.e., C_max_ and AUC for S-PE-SNEDDS (SOF3), were found to be significantly higher (*p* < 0.05) than that of pure PE-dispersion (Table 7). The value of Tmax, half-life, and elimination rate constant for S-PE-SNEDDS (SOF3) was found to be 2 h, 7.23 h ± 0.39, and 0.096 h^−1^ ± 0.006, respectively, as compared to pure PE dispersion, which exhibited 1 h, 5.81 ± 0.57, and 0.11 ± 0.009 h^−1^, respectively. Cmax for S-PE-SNEDDS (SOF3) was around 3.33-fold higher than that of pure PE dispersion. The AUC and oral bioavailability of PE from optimized S-PE-SNEDDS (SOF3) was 4.92-fold higher than that of pure PE dispersion. The significant enhancement in the bioavailability might be due to nano particle size as well as due to higher solubility.

#### 3.12.3. Histological Examination

**Figure 12:**
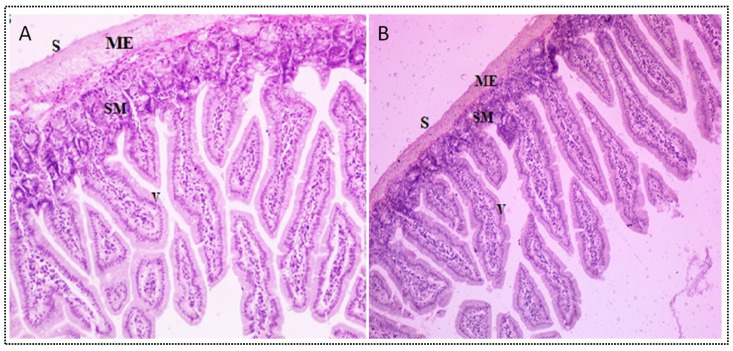
Histopathology image: (**A**) intestine section of normal albino rat, (**B**) showing: Serosa (S), muscularis externa (ME), submucosa (SM) with numerous eosinophilic granule-containing cells (rectangle), and mucosa (M) with epithelial layer and villi (V).

Figure 12A,B shows the structure of the intestinal lumen of the control and PE-S-SNEDDS-treated rats. The control intestinal membrane showed normal serosa, muscularis externa, and submucosa along with the numerous eosinophilic granule-containing cells (rectangle) and mucosa with epithelial layer and villi. There was a similar observation also found with the S-PE-SNEDDS (SOF3)-treated group. The internal structure was found to be similar. There is no inflammation of the intestinal membrane. The inclusion of PE in SNEDDS might be safer due to the prevention of direct contact with intestinal mucosa with PE.

### 3.13. Stability Study

The parameter of S-PE-SNEDDS (SOF3) (globule size, % transmittant, and emulsification) was evaluated at accelerated condition, and the result showed non-significant (*p* > 0.05) changes in all parameters. The Arrhenius plot was constructed and the degradation rate constant (K_25°C_) was found to be 1.77 × 10^−4^/month. The shelf-life of S-PE-SNEDDS (SOF3) was calculated and found to be 1.62 years.

## 4. Conclusions

PE-loaded SNEDDS was prepared and optimized by Box Behnken design. The optimized PE-SNEDDS (OF3) was transformed into S-PE-SNEDDS by adsorbing it on porous avicel PH-101. The PE-SNEDDS (OF3) and S-PE-SNEDDS (SOF3) exhibited acceptable in-vitro characteristic parameters. S-PE-SNEDDS (SOF3) displayed good thermodynamic stability, quick self-emulsification, and better in-vitro release with enhanced bioavailability. The pharmacodynamic S-PE-SNEDDS (SOF3) showed a better effect against hypertension than pure PE. The formulation also exhibited pronounced antibacterial activity as well as in-vitro anti-oxidant activity. Finally, it can be concluded that a solid self-nanoemulsifying drug delivery system of PE could be a suitable and finer approach for the enhancement of dissolution, bioavailability, and therapeutic efficacy.

## Figures and Tables

**Table 1:** Variables for Box-Behnken design optimization.

Formulation Variables	Level (Coded Value)		
	**Low (−1)**	**Medium (0)**	**High (+1)**
Oil (%)	15	37.5	60
Surfactant (%)	25	47.5	70
Co-surfactant (%)	10	25	40
**Responses**	**Goal**		
Globule size (nm)	Minimum		
Transmittance (%)	Maximum		
Emulsification time (Second)	Minimum		

**Table 2:** Formulation composition for various composition obtained from Box-Behnken design with actual and predicted values of responses.

Code	Oil Concentration (%)	Conc of Surfactant (%)	Conc of Co-Surfactant (%)	Globule Size (nm)		Transmittant (%)		Emulsification Time (S)	
				Actual Value *	Predicted Value	Actual Value *	Predicted Value	Actual Value *	Predicted Value
F1	15	25	25	62.03 ± 3.54	63.05	97.28 ± 1.23	97.17	59 ± 2	60
F2	60	25	25	200.34 ± 4.23	201.70	85.23 ± 2.42	85.22	144 ± 3	143
F3	15	70	25	30.52 ± 5.12	28.30	98.00 ± 2.12	98.01	46 ± 2	48
F4	60	70	25	117.91 ± 3.65	116.89	96.15 ± 3.65	96.26	81 ± 2	80
F5	15	47.5	10	65.54 ± 5.61	66.14	93.30 ± 1.42	93.37	62 ± 3	63
F6	60	47.5	10	178.78 ± 5.43	179.24	89.10 ± 1.23	89.07	135 ± 2	136
F7	15	47.5	40	25.16 ± 6.17	24.70	99.89 ± 2.46	99.92	33 ± 3	32
F8	60	47.5	40	140.54 ± 5.32	138.86	90.57 ± 1.76	90.50	75 ± 2	77
F9	37.5	25	10	170.76 ± 7.43	167.84	88.54 ± 1.27	88.58	106 ± 2	107
F10	37.5	70	10	95.23 ± 6.76	95.56	96.23 ± 1.65	96.15	42 ± 2	43
F11	37.5	25	40	115.43 ± 3.65	114.44	94.12 ± 1.61	94.20	35 ± 2	35
F12	37.5	70	40	65.65 ± 3.87	67.16	98.56 ± 1.46	98.52	23 ± 3	24
F13 *	37.5	47.5	25	109.21 ± 6.32	103.12	97.56 ± 2.43	97.79	62 ± 2	62
F14 *	37.5	47.5	25	101.54 ± 5.32	103.54	97.98 ± 1.43	97.79	61 ± 3	62
F15 *	37.5	47.5	25	104.24 ± 3.54	103.76	97.76 ± 1.65	97.79	63 ± 2	62
F16 *	37.5	47.5	25	100.76 ± 5.36	103.63	97.76 ± 1.67	97.79	63 ± 3	62
F17 *	37.5	47.5	25	101.71 ± 4.58	103.21	97.89 ± 1.87	97.79	62 ± 2	62

* Centre point (same composition, values are mean ± SD, (*n* = 3).

**Table 3:** Model statistical summary of best-fitted quadratic model of each response.

Statistical Term	Globule Size (Y_1_)	% Transmittant (Y_2_)	Emulsification Time (Y_3_)
R^2^	0.9980	0.9995	0.9996
Adjusted R^2^	0.9954	0.9988	0.9991
Model F-Value	388.10	1476.83	1936.34
Model *p*-value	<0.0001	<0.0001	<0.0001
Lack of fit F-value	0.51	0.67	0.39
Lack of fit *p*-value	0.6945 *	0.6129 *	0.7693 *
Adequate Precision	70.594	130.299	169.586

* Represent the non-significant.

**Table 4:** Point prediction optimization of SNEDDS from software.

Code	Composition of SNEDDS	Actual Value			Predicted Value		
	Oil	Globule Size (nm)	Transmittant (%)	Emulsification Time (Sec)	Globule Size (nm)	Transmittant (%)	Emulsification Time (Sec)
OF1	37.50:47.50:25.00	104.23 ± 2.54	97.87 ± 2.12	61 ± 1	103	97.79	62
OF2	30.00:46.28:25.00	86.54 ± 3.65	98.65 ± 2.17	55 ± 2	84.93	98.55	56
OF3	25.00:46.28:25.00	70.34 ± 3.27	99.02 ± 2.02	53 ± 2	71.21	98.87	54

Oil (GML, %): surfactant (% Poloxamer 188, %): Co-surfactant (Transcutol HP, %).

**Table 5:** Composition of optimized S-PE-SNEDDS (SOF3).

Ingredients	Concentration (mg)	Composition (%)
Piperine	20	7.51
GML	25	9.39
Poloxamer 188	46.28	17.38
Transcutol HP	25	9.39
Avicel	150 mg	56.33

**Table 6:** Release kinetic model applied to evaluate the best-fitted model for S-PE-SNEDDS (SOF3).

S.No.	Kinetic Model	Plot	R^2^
1	Zero order	% cumulative amount of drug released versus time	0.9891
2	First order	Log % cumulative drug remaining versus time	0.8142
3	Higuchi’s model	% cumulative amount of drug released versus square root of time	0.9631
4	Korsmeyer-Peppas model	Log of fraction of drug released/ Log cumulative % drug released versus log time	0.9352 *n* = 0.2096

**Table 7:** Pharmacokinetic parameters of pure PE-dispersion and S-PE-SNEDDS after single-dose oral administration. Values are given as mean ± SD (*n* = 6).

Parameters	Pure PE	S-PE-SNEDDS
Cmax (µg/mL)	149.18 ± 25.29	585.14 ± 39.47
Tmax (h)	1	2
Ke (h^−1^)	0.11 ± 0.009	0.0958 ± 0.006
T_1/2_ (h)	5.81 ± 0.57	7.23 ± 0.39
AUC_0–12_ (µg·h/mL)	667.14 ± 27.39	3718.99 ± 83.72
AUC_0–α_ (µg·h/mL)	866.14 ± 43.91	5177.43 ± 157.36

## Data Availability

Not applicable.
